# General practitioners in German metropolitan areas – distribution patterns and their relationship with area level measures of the socioeconomic status

**DOI:** 10.1186/s12913-016-1921-5

**Published:** 2016-11-25

**Authors:** Jan Bauer, Doerthe Brueggmann, Daniela Ohlendorf, David A. Groneberg

**Affiliations:** Institute of Occupational, Social and Environmental Medicine, Goethe University, Theodor-Stern-Kai 7, 60329 Frankfurt/Main, Germany

**Keywords:** Primary care, Access, Distribution, Urban, Socioeconomic status

## Abstract

**Background:**

Geographical variation of the general practitioner (GP) workforce is known between rural and urban areas. However, data about the variation between and within urban areas are lacking.

**Method:**

We analyzed distribution patterns of GP full time equivalents (FTE) in German cities with a population size of more than 500,000. We correlated their distribution with area measures of social deprivation in order to analyze preferences within neighborhood characteristics. For this purpose, we developed two area measures of deprivation: Geodemographic Index (GDI) and Cultureeconomic Index (CEI).

**Results:**

In total *n* = 9034.75 FTE were included in *n* = 14 cities with *n* = 171 districts. FTE were distributed equally on inter-city level (mean: 6.49; range: 5.12–7.20; SD: 0.51). However, on intra-city level, GP distribution was skewed (mean: 6.54; range: 1.80–43.98; SD: 3.62). Distribution patterns of FTE per 10^4 residents were significantly correlated with GDI (*r* = −0.49; *p* < 0.001) and CEI (*r* = −0.22; *p* = 0.005). Therefore, location choices of GPs were mainly positively correlated with 1) central location (*r* = −0.50; *p* < 0.001), 2) small household size of population (*r* = −0.50; *p* < 0.001) and 3) population density (*r* = 0.35; *p* < 0.001).

**Conclusion:**

Intra-city distribution of GPs was skewed, which could affect the equality of access for the urban population. Furthermore, health services planners should be aware of GP location preferences. This could be helpful to better understand and plan delivery of health services. Within this process the presented Geodemographic Index (GDI) could be of use.

## Background

Discrepancies in access, supply and demand of primary care constitute an intensified focus of health care legislation worldwide. Many European countries face a shortage of general practitioners (GPs), e.g. for the UK, an additional need of approximately 8000 full-time GPs is projected by 2020 [1]. Furthermore, the occupational profile of GPs is perceived as unattractive based on suboptimal salaries, administrative burdens and an increasing complexity of care [2]. Thus, many junior physicians pursue training in other specialties [2]. However, several solutions have been proposed to this shortage without the need for more trained GPs. These solutions mainly focused on the empowerment of non-physician workforce [3, 4].

In addition to decreasing absolute provider numbers, relative numbers in terms of the spatial distribution of the GP workforce vary geographically. This spatial distribution has been shown to depend on numerous socioeconomic and demographical factors: Large demographic studies identified a shortage of providers located in the countryside and a relative oversupply in metropolitan areas leading to a mismatch of demand and supply [5, 6]. Similar to the GP distribution (supply), population sizes (demand) vary geographically: people are migrating towards large urban centers to pursue job opportunities or further education [7]. In addition, the populations’ socioeconomic status (SES) as a major determinant of health inequity has also been shown to vary geographically [7]. Furthermore a low SES has been linked to a poor health status with a higher rate of overall mortality [7–10]. In particular, morbidities such as cardiovascular events, stroke or diabetes are more common among the socially deprived population [11–13].

In order to manage local provider shortages in European countries, spatial distribution of GPs has been regulated [6]. In Germany, administrative areas (planning areas) have been installed for which the number of GPs is restricted. This restriction takes effect if a population-to-GP ratio of 1:1671 is exceeded by 10% [14]. However, within these restricted administrative areas, a GP can choose his or her preferred practice location. Since measurements based on population-to-provider ratios (PPR) have limitations, the current capacity plan has become subject of recent discussions [15]. In this context, little is known about the factors associated with settlement decisions of GPs in metropolises. So far, numerous studies have focused on urban vs. rural comparisons of GP distribution and their impact on health inequity [16–22]. Only few studies have investigated metropolitan areas in Germany [23, 24]. Therefore, the objectives of this study were (1) to compare spatial distribution of GPs on intra- and inter-city level, (2) to relate the distribution pattern to the geo-social environment defined by the populations’ SES and (3) to extract urban location choice preferences of GPs.

## Methods

**Fig. 1:**
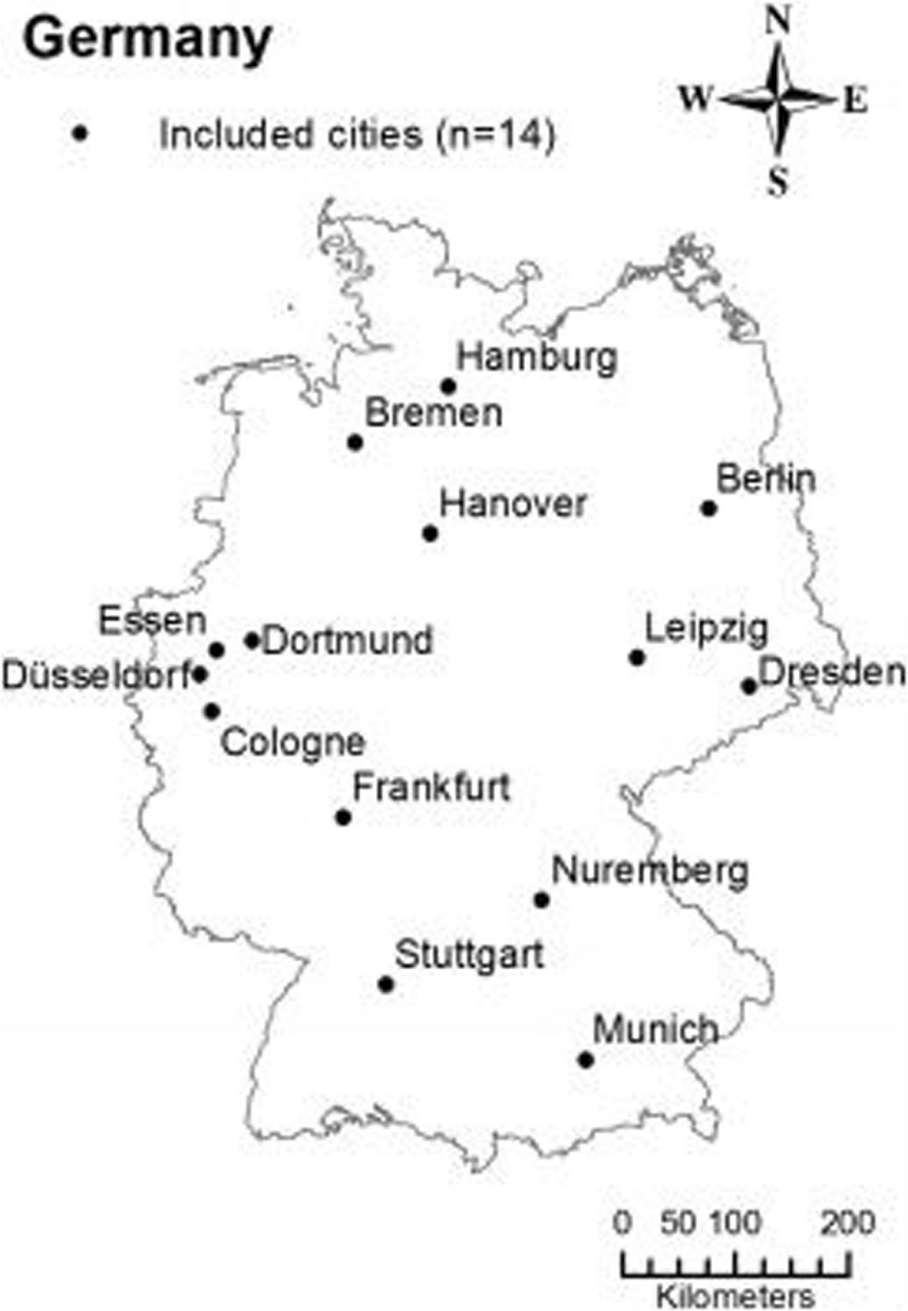
Included cities (*n* = 14) in Germany. This figure is a derivative of geographic data provided by the “Federal Agency for Cartography and Geodesy” © GeoBasis-DE/BKG 2013. The permission to use and adapt this figure is stated in the “GeoNutzV (§2)” [45]

In this study, we investigated 14 German metropolises with a population size of more than 500,000 according to Census data from 2013. Thus, the following cities were included (in declining population size order): Berlin, Hamburg, Munich, Cologne, Frankfurt, Düsseldorf, Stuttgart, Dortmund, Essen, Leipzig, Bremen, Dresden, Hanover and Nuremberg. All cities are shown in Fig. 1.

The analysis was performed on the largest-scale city division, for which statistical data were available. These were defined as city districts (total of *n* = 171 districts in *n* = 14 cities). The total population size for all districts was *n* = 13,638,160.

### Data sources and measures of outcome variables

*GP Data*: Data were obtained from the National Association of Statutory Health Insurance Physicians in Germany (KBV). GPs were defined as physicians actively participating in primary care. According to § 11 (2) of the ‘directive of capacity planning’ by the Federal Joint Committee, physicians of the following medical specialties can account as GPs: 1) family medicine, 2) internal medicine and 3) physicians without specialization. The number of GPs was measured by their full time equivalent (FTE) in regard to their contracted participation in the delivery of primary care (§ 21 and § 22 ‘directive of capacity planning’). Therefore, the range of FTE of a single GP can range between 0 (no participation) and 1 (full participation) with steps of 0.25. The number of FTE per district was obtained from the KBV.

*Geographic data*: City district boundaries were retrieved from communal administrative bureaus. Additionally, the internet-based tracking software GPSies [25] in combination with official district maps has been used for heads-up digitizing. City district boundaries are created and regularly revised by local authorities due to political and/or geographical changes. We applied city district boundaries as of 2013. Furthermore, coordinates of each city center (*n* = 14) were retrieved via Google Maps (Google Inc., Mountain View, USA). The city center was defined as the respective city hall.

*Socioeconomic status (SES)*: Indicator data of SES were retrieved from official city statistics. We obtained the latest data available as of 2015 (oldest data as of 2010; mostly 2014). In total, *n* = 10 indicator variables were used (see Table 1). These variables were chosen based on their theoretical influence on the SES and their availability on city district level.

**Table 1:** Demographic data and SES indicators

Indicator	Description	Unit
Population density	Number of residents per km^2^	*n*/km^2^
Distance to city hall	Airline distance to the city hall	m
Old-age dependency ratio	Number of residents over 65 years per 100 residents aged 15–64 years	%
Migrant quota	Quota of residents with migration background to all residents (migration background was defined as foreign citizenship *or* dual citizenship *or* background of parental foreign citizenship)	*n*
Household size	Average number of residents per household	*n*
Employment quota	Quota of employed residents subject to social insurance contribution to all residents aged 15–64	%
Unemployment quota	Quota of unemployed residents to all residents aged 15–64	%
Benefits recipients quota	Quota of unemployed residents aged 15–64 receiving state subsidy to all residents aged 15–64	%
Motorization rate	Number of privately used automobiles per 1000 residents	*n*
Mortality	Number of deaths per 1000 residents	*n*

### Calculations and statistical analysis

Data of GPs and population sizes on district level were used to build population-to-provider ratios (PPR) as measures of spatial distribution (PPR = FTE per 10^4 residents) [16]. In addition, linear airline district-city-center distances were calculated (from mean district coordinates to city hall coordinates) with the software QGIS (QGIS Development Team, General Public License). We standardized all measures by computing z-scores individually for each city. Hereby, inter-city district data were comparable.

### Development of SES composite indices

A principal component analysis (PCA) was performed with all *n* = 10 SES indicators to create composite indices. Eligible indicators were tested by using the measure of sample adequacy (Kaiser-Meyer-Olkin) and the Bartlett’s test of sphericity. The composite indices represented area measures of SES. According to PCA results, index scores were calculated with a regression method (scores have a mean of 0; variance is equal to the squared multiple correlation between estimated score and true value). Again, z-scores were computed to standardize inter-city comparison. Furthermore, a correlation analyses (Spearman’s Rho) was performed using SPSS 23 (IBM, Armonk, USA). For the correlation, we applied the following definitions:

- *r* < |0.2|: no relevant correlation.
- |0.2| ≤ *r* < |0.3|: weak correlation.
- |0.3| ≤ *r* < |0.5|: mild correlation.
- |0.5| ≤ *r* < |0.7|: strong correlation.
- *r* ≥ |0.7|: very strong correlation.

We further performed a multiple linear regression analysis. Prior, we also tested for its assumptions: 1) linearity, 2) normality, 3) homoscedasticity, 4) independence of observations, 5) multicollinearity and 6) assumptions according to the Gauss–Markov theorem. Model assumptions were controlled for visually (boxplot and histogram), with the Durbin-Watson test and the variance inflation factor.

## Results

In total *n* = 9034.75 FTE were located within boundaries of the 14 metropolitan cities. However, in 10 out of 171 districts the number of FTE was less than five and due to data protection, the exact number could not be obtained. For statistical reasons the number of FTE for these districts was set to *n* = 2.5. An overview of the cities and the number of FTE is shown in Table 2.

**Table 2:** Overview of cities, population and FTE

Metropolitan cities (number of districts)	Population	Population Density	FTE			
			∅ FTE/district	residents/FTE	PPR (SD)	Supply level
	(*n*)	(*n*/km^2^)	(*n*)	(*n*)	(*n*)	(%)
Berlin (*n* = 12)	3,562,166	3995	198	1496	6.68 (1.07)	120
Hamburg (*n* = 7)	1,788,994	2369	176	1449	6.90 (0.80)	118
Munich (*n* = 25)	1,490,678	4797	43	1388	7.20 (7.78)	122
Cologne (*n* = 9)	1,053,528	2602	79	1486	6.73 (2.14)	116
Frankfurt (*n* = 16)	693,342	2792	28	1526	6.55 (2.37)	119
Düsseldorf (*n* = 10)	603,210	2784	40	1494	6.69 (2.10)	115
Stuttgart (*n* = 23)	592,898	2863	16	1594	6.27 (3.30)	105
Dortmund (*n* = 12)	589,283	2099	25	1955	5.12 (1.41)	111
Essen (*n* = 9)	576,691	2805	38	1700	5.88 (1.07)	124
Leipzig (*n* = 10)	551,870	1854	37	1512	6.61 (1.37)	110
Bremen (*n* = 4)	548,547	1726	74	1488	6.72 (3.31)	112
Dresden (*n* = 10)	541,304	1649	33	1619	6.18 (0.94)	102
Hanover (*n* = 13)	528,879	2591	27	1524	6.56 (2.65)	113
Nuremberg (*n* = 10)	516,770	2771	35	1474	6.78 (2.38)	117

“Supply levels” describe the official supply of GPs (in %) for each city as calculated by the KBV [46] as of 2015 (Geographical base of calculating supply levels differed from statistic boundaries used in this study). ∅: city average. *FTE* full time equivalent in regard to their contracted participation in primary care, *PPR* FTE per 10^4 residents (see Methods section for further details). *SD* Standard Deviation

**Fig. 2:**
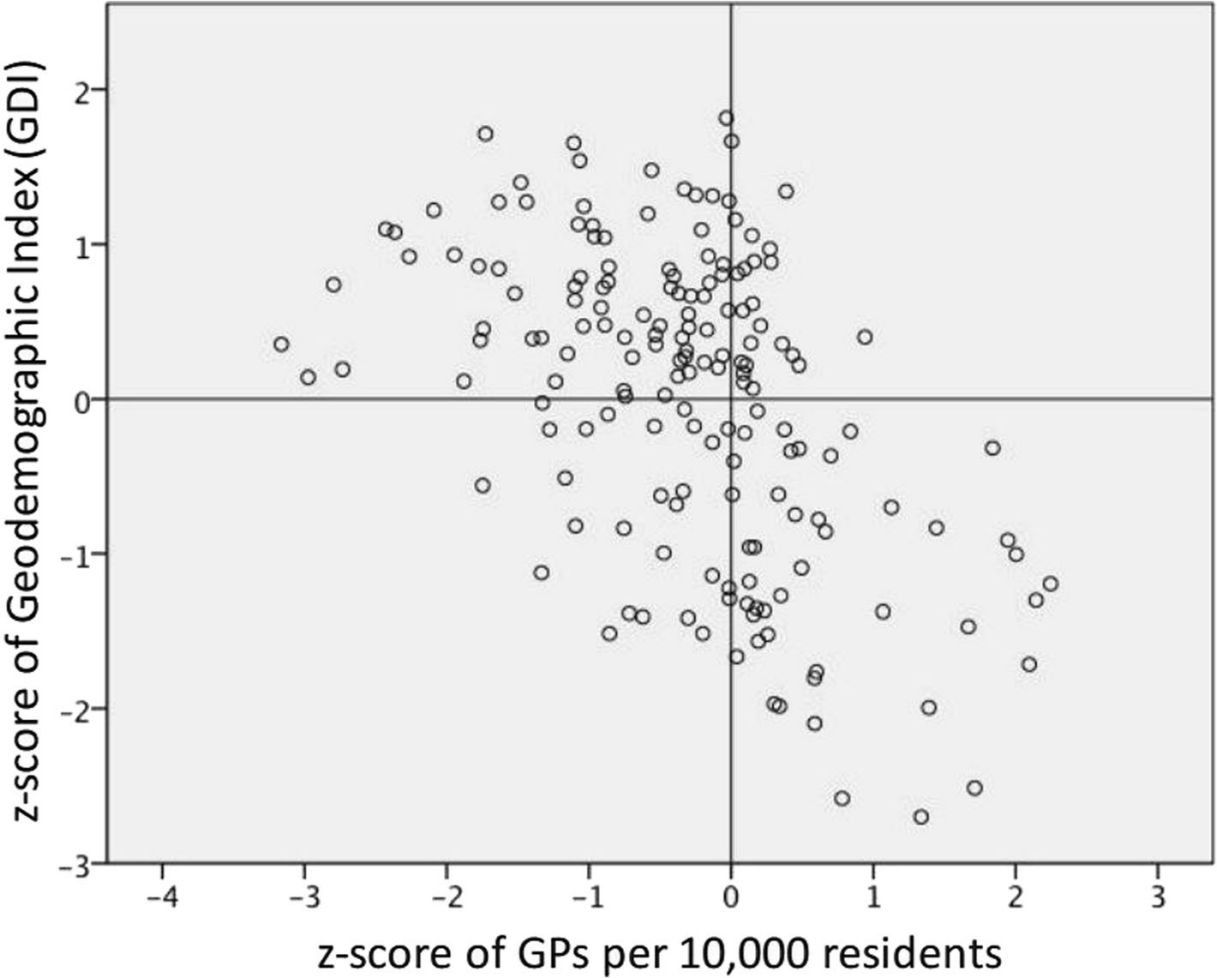
Residents per full time equivalent (FTE) for districts in Berlin (*n* = 12) and Frankfurt (*n* = 16). This figure is a derivative of “RBS-Blöcke, Dezember 2015” by “Amt für Statistik Berlin-Brandenburg” used under license CC BY 3.0 DE and “Frankfurter Stadtteilgrenzen für GIS-Systeme” by “Bürgeramt, Statistik und Wahlen” used under license dl-de/by-2-0

On city level, spatial distribution of GPs in terms of FTE per 10^4 residents (PPR) was homogenous with a mean PPR of 6.49 (range: 5.12–7.20; SD: 0.51). However, on district level inhomogeneous spatial distribution was present with a wider range of PPRs (range: 1.80–43.98) and a greater standard deviation (SD: 3.62). Still, the mean PPR was similar with 6.54. Looking at individual cities, the analysis revealed a differing city district variation: A high variation was present in Munich (SD: 7.78) whereas low variation was present in Hamburg (SD: 0.80). In Fig. 2, the spatial distribution of GPs on district level is shown for Berlin and Frankfurt. In conclusion, unlike inter-city distribution, the intra-city distribution was inhomogeneous.

### Results of PCA

In total, *n* = 10 SES indicators were tested eligible for the principal component analysis (PCA). Visualization of scree plot suggested a two-component solution. Two variables with communalities after extraction of <0.4 were dismissed: ‘Mortality’ (communality x = 0.267) and ‘employment quota’ (communality x = 0.128). PCA was repeated with *n* = 8 indicators resulting in two composite area measures of SES named (1) Geodemographic Index (GDI) and (2) Cultureeconomic Index (CEI). Results of the PCA are displayed in Table 3.

**Table 3:** Results of PCA

Indicator	Index loading		Index coefficient	
	GDI	CEI	GDI	CEI
Population density	**−0.809**	0.047	−0.242	−0.040
Distance to city hall	**0.909**	−0.044	0.273	0.049
Old-age dependency ratio	**0.742**	−0.192	0.212	−0.018
Migrant quota	−0.225	**0.860**	−0.007	0.301
Household size	**0.869**	0.121	0.272	0.107
Unemployment quota	−0.058	**0.962**	0.051	0.350
Benefits recipients quota	0.029	**0.970**	0.078	0.360
Motorization rate	**0.781**	−0.423	0.207	−0.100

Index loading is based on a rotated component matrix (rotation method: Varimax with Kaiser normalization; bold numbers indicate high loading of indicator in index). Index coefficient was based on the component score coefficient matrix (coefficients by which indicators were multiplied to build GDI and CEI). *PCA* principal component analysis, *GDI* geodemographic index, *CEI* cultureeconomic index

GDI was mainly loaded with the following indicators: population density, distance to city hall, old-age dependency ratio, household size and motorization rate. CEI was mainly loaded with migrant quota, unemployment quota, and benefits recipients’ quota. The total variance explained by both indices was 78.6%. After rotation GDI explained 43.1% and CEI 35.5% of total variance.

### Correlation of area measures of SES and spatial distribution of FTE

**Fig. 3:**
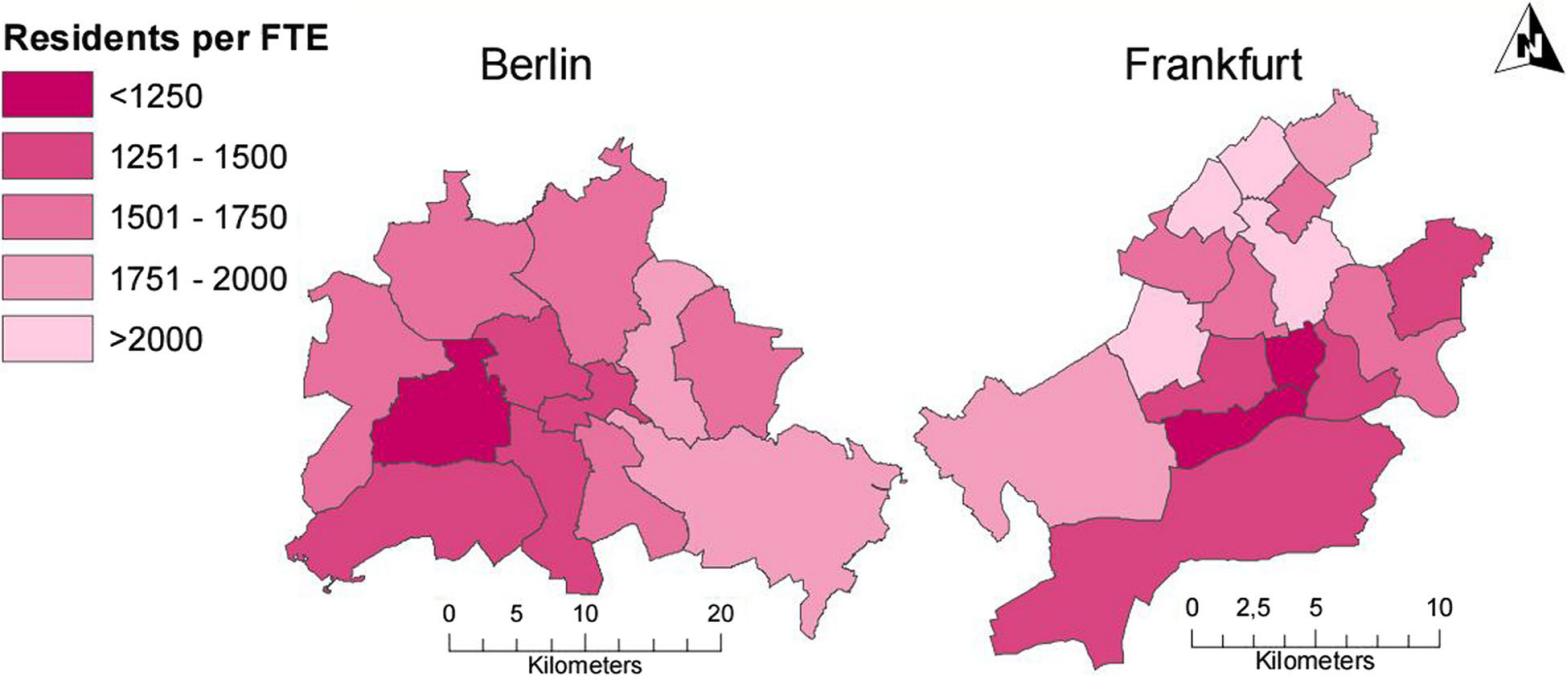
Scatter plot of districts (*n* = 171) in regard to z-scores of GDI and GP density. *GDI* geodemographic index, *GP* weighted capacity planning number of general practitioners

As seen in Fig. 3, a significant mild negative correlation was present between the spatial distribution of FTE and the Geodemographic Index (GDI) with a negative correlation of *r* = −0.49 (*p* < 0.001). Considering the indicators comprising GDI, we ranked the indicators that were positively correlated with the number of FTE in the respective district in declining order:

1. Central location (indicator: distance to city hall).
2. Small household size (indicator: household size).
3. Crowded district (indicator: population density).
4. Low motorization rate (indicator: motorization rate).
5. Young district (indicator: old-age dependency ratio).

In regard to CEI, a significant negative correlation (*r* = −0.22; *p* = 0.005) was found. This result indicated that CEI can explain GP settlement decisions to a lesser extent compared to GDI. Still, focusing on the indicators comprising CEI, we can deduce that a low migrant quota, low unemployment quota, and low benefits recipient quota has potentially positive effects on the number of FTE per district.

These findings were confirmed by a correlation analysis (see Table 4): The indicators “mortality” and “employment quota”, which were excluded by the PCA, showed no significant correlation with the spatial distribution of GPs (PPR). Furthermore, a correlation of *r* = −0.50 (*p* < 0.001) for both “distance to city hall” and “household size” with PPR was revealed.

**Table 4:** Correlation and regression analysis of PRR with measures of SES

		Correlation analysis		Regression analysis	
		*r*	*p*-value	beta	*p*-value
PPR	GDI	*−0.49*	*<0.001*	–	–
	CEI	*−0.22*	*0.005*	–	–
PRR	population density	*0.35*	*<0.001*	−0.11	0.244
	distance to city hall	*−0.50*	*<0.001*	*−0.31*	*0.012*
	old age dependency ratio	*−0.24*	*0.002*	0.16	0.077
	migrant quota	−0.03	0.702	–	–
	household size	*−0.50*	*<0.001*	*−0.30*	*0.006*
	employment quota	0.01	0.880	–	–
	unemployment quota	*−0.20*	*0.011*	–	–
	benefits recipients quota	*−0.24*	*0.002*	*−0.24*	*0.002*
	motorization rate	*−0.32*	*<0.001*	−0.20	0.062
	mortality	−0.10	0.180	–	–

Correlation (Spearman’s rho; *r*) was computed for z-scores. *GDI* geodemographic index, *CEI* cultureeconomic index, *PPR* FTE per 10^4 residents, *SES* socioeconomic status

Italic entries are significant with *p*<0.05

We further performed a multilevel regression analysis allowing for multi-membership (Table 4). We included all indicators that showed a relevant correlation (*r* > 0.2) in the correlation analysis (*n* = 6). The analysis revealed that the regression model was significant (F-test: *p* < 0.001) and showed no relevant multicollinearity (Durbin-Watson test: 2.247). The corrected coefficient of determination was R^2^ = 0.332, which showed that 33% of the PPR variability has been accounted for. Therefore, the Cohens’ effect size was f^2^ = 0.70, which indicated a strong effect. The regression analysis supported the finding that the distance to the city hall was the most important factor affecting the number of FTE per 10^4 residents on district level, followed by household size and benefits recipients’ quota.

## Discussion

The results of this study suggest that GPs are *unequally* distributed on inner-city level and *equally* distributed on inter-city level in German metropolitan areas with more than 500,000 residents. On district level, we documented that the distance to the city hall was the most important factor explaining higher GP densities, followed by household size and benefits recipients’ quota. In this regard, the presented Geodemographic Index (GDI) could be used for planning purposes of GP health care services in urban areas.

### GP distribution on intra-city level

We documented an unbalanced spatial distribution of GPs in terms of GP density in German metropolises on intra-city level. This finding is in line with current literature: A study conducted within Adelaide (Australia) found similar distribution patterns: 16% of residents were reported to live in an area of GP shortage within Adelaide boundaries [26]. An unbalanced distribution of physicians was further reported for Auckland (New Zealand) [27] and Toronto (Canada), where the authors reported a range of 6.18–12.42 GPs per 10^4 residents for the city districts of Toronto [28]. In our study, the number of FTE per 10^4 residents ranged between 1.80–43.98 and therefore variation was higher compared to the Canadian study. However, we included 14 cities whereas the aforementioned studies only reported results from individual cities, which increased the likelihood of data variability. Still, our results can be considered more valid due to the bigger sample size.

Furthermore, the extent of the demonstrated distribution varied between the analysed cities. For example, the high variation in Munich could be due to a greater competition between GPs, which translated into higher GP densities near the city centre and vice versa. However, it has to be noted that the number of districts varied between the cities and especially the number of districts in Munich used in this study was higher compared to all other cities. This could explain the higher variation in Munich.

### GP distribution on inter-city level

On inter-city level, the distribution of GP densities was balanced. In other words, there was only little variability in GP density data. We assume that this balanced GP distribution pattern was due to strict German health care regulations, which restrict GP numbers in certain areas such as larger cities. Such areas are defined “oversupplied” as soon as the population-to-GP ratio exceeds 1:1671 by 10% (§ 11 (4) of the ‘directive of capacity planning’). In this case, GPs will not receive permission to establish practices in these areas.

In general, studies have shown that GPs as well as other medical specialties tend to be located in urban areas [5, 6]. The data presented in our study revealed higher GP densities on city level: in our study the overall physician-to-population ratio (PPR) was 1:1550 (SD: 139.3). Compared to the aforementioned threshold value of 1:1671 (the ‘optimum ratio’) the ratio was exceeded by 7.8%. Therefore, applying the definition of oversupply mentioned above, these cities cannot be considered oversupplied. However, it has to be noted that within national capacity planning, adjustments can be made according to demography and/or geography. Regarding the non-physician participation in primary care, they were shown to constitute the smallest share of primary care workforce in urban areas and were more likely to delivery primary care in rural areas [29]. Therefore, lack of primary care services in more rural areas could be compensated by non-physician workforces. This solution to GP workforce shortage has also been reported by recent literature [3, 4].

In addition to the aforementioned variation of district numbers, differing district sizes could conceal further variation. However, city district sizes and numbers are not standardized in Germany, which limits the inter-city comparison. Therefore, the heterogeneity of district sizes and numbers could have concealed inter-city variations.

### GP location choices

Using socioeconomic indicators, a correlation with GP densities was present that could explain GP location choices on city district level. In regard to the GDI, a mild negative correlation was present (*r* = −0.49) indicating a negative influence on GP location choices. Looking at the factors comprising GDI, the most important factor was a spatial factor (‘distance to the city hall’). Therefore, our results suggested spatial factors to be more important than non-spatial factors. However, in a survey [23] among 117 physicians in Germany, 81% of participants stated that neighborhood characteristics concerning the suitability for children and families was the most important factor regarding practice location choice. The high impact of the ‘significant other’ on GP location choices has also been reported by Smith et al. [30]. Furthermore, spatial factors such as ‘proximity to work place of significant other’ or ‘proximity to city center’ were reported to only play a minor role regarding GP location choices [23]. However, a study in Finland [31] further reported ‘location of workplace’ and ‘being near a central hospital’ as the 2nd and 6th most important factor for the GPs workplace choice. In addition, proximity to other health care providers (e.g. dentist, pharmacies, hospitals) was further reported to influence location choices of GPs [31, 32]. In our study, pharmacies, dentists, hospitals and the work place of GP spouses were not measured and therefore not controlled for. In summary, it can be stated that spatial factors as reported in our study are important factors influencing the practice location choice of GPs. However, taking further research into account, non-spatial factors seem to have an even greater impact compared to spatial factors. The non-spatial factors comprising the GDI, namely household size and population density, were also used as high loading factors in the urban index (UX) created by Schulz et al. [24]. In their study, no relevant correlation (*r* = −0.13) between UX and the demand for GP services was present. Considering both UX and GDI, the reported findings suggested that household size and population density influence the GP practice location choice (i.e. provider supply), but have no influence on the populations’ demand for health care services. Looking at other non-spatial factors, Gosden et al. [33] reported ‘aversion to location in areas of high deprivation’ as the most important factor influencing GP practice location choices. In our study, deprivation was mostly reflected by CEI. However, a weak correlation (*r* = −0.22) was found, which suggests only a minor influence on practice location choices. Further factors include financial incentives often represented by the percentage of the population with private health insurance, which has been shown to explain 14% of the GP density variation in Germany, while ‘health care needs’ only explained less than 5.2% [34].

It has to be noted that location choices of GPs can be influenced by both external and internal factors. Internal factors such as personal characteristics have been shown to play an important role in the workplace selection process [35]. However, in our article we did not control for internal factors and focused solely on external factors defined by neighborhood statistics.

In regard to mortality, our study found no correlation with the spatial distribution of GPs. This finding is in line with current literature which showed no significant difference of mortality (deaths per 100 beneficiaries) in regard to differing GP densities [36].

Finally, many countries limit the GPs’ practice location choice in order to sustain equal access, regardless of a patients’ living location. The degree of this limitation usually depends on the performance of the health care system in place: The more sophisticated the health care system, the more regulations are in place, which in turn limits the GPs practice location choices. China for example has a rather weak primary care system, whereas the United Kingdom and Germany have a rather strong primary care system [6, 22]. Bearing this in mind, our results only reflect the situation in a developed health care setting.

Although GPs do not fulfill a formal compulsory gatekeeper function in Germany, their availability and accessibility play a crucial role in a populations’ access to health care [6, 37]. Hence, it can be assumed that an unbalanced distribution of GPs could translate into health inequities [38, 39]. A mismatched distribution of GPs constitutes a major challenge for public and community health. Thus, tailored strategies and policies are desperately needed to address these challenges.

### Limitations

Population-to-provider ratios (PPR) have limitations in regard to the measurement of spatial accessibility [16, 40]. As outlined by Guagliardo, limitations of PPR include 1) not accounted border crossing, 2) blindness to variabilities within bordered areas, 3) omission of distance/time and 4) fixation to geographical/administrative boundaries [16]. Especially the omission of border crossing in the presented approach represents a limitation. More sophisticated measurements, which addressed these issues, are based on gravity models such as the Two Step Floating Catchment Area (2SFCA) method [41]. Since in Germany the population-to-provider ratio is still widely used in research and in the political context, we used a PPR despite the known limitations. However, we acknowledge the great value of more sophisticated measures using geographic information systems. Furthermore, using airline distances instead of time distances, represents a distance measurement simplification and therefore a loss of accuracy must be assumed. However, airline distances have also been used in similar research [21].

In addition to the aforementioned limitations, there are four limitations that have to be further addressed: First, there is no best indicator for measuring SES. Residual confounding by unmeasured socioeconomic factors can only be minimized, not excluded. On area level, different single measurements or composite indicators have been established (e.g. English Index of Multiple Deprivation [42], Bavarian Index of Multiple Deprivation [43], Townsend Deprivation Index). However, due to insufficient data availability, none of the described composite indicators could be used for the nationwide inner-city analysis in Germany.

Furthermore, city district data were heterogeneous and therefore the accuracy of the results could be compromised. In regard to GP practice locations, the allocation to city district boundaries has limitations due to possible errors retrieving geo coordinates [44].

## Conclusion

Intra-city distribution of GPs was skewed, which could affect the equality of access for the urban population. Furthermore, health services planners should be aware of location preferences of GPs within major urban conurbations. This could be helpful to understand and plan the delivery of health care services. For this process the presented Geodemographic Index (GDI) could be of use.

## Abbreviations

CEI: Cultureeconomic Index
GDI: Geodemographic Index
GP: General Practitioner
KBV: National Association of Statutory Health Insurance Physicians
PCA: Principal component analysis
SES: Socioeconomic status
UK: United Kingdom
